# Supplementary material for: A Novel Tool to Assess the Risk for African Swine Fever in Hunting Environments: The Balkan Experience
Source: Pathogens. 2022 Dec 3;11(12):1466. doi: 10.3390/pathogens11121466 (PMC9787848; doi:10.3390/pathogens11121466)
Supplement: Supplementary file 1 [file pathogens-11-01466-s001.zip › Supplementary File S1.pdf]

Biosafety List for Hunting Grounds

Survey

1. The questionnaire should be conducted by an interviewer, rather than just leaving it with the interviewed person. 2. It takes around 30 minutes to fill in one questionnaire. 3. When asking the questions, it is better to wait for the interviewed person to answer and then look for the answer in the list of options, rather than reading all the options to them, thus potentially misguiding their answers. 4. Please turn on your GPS on your deviceand enable epicollect5 to use the phone's GPS and camera.

Hunting Grounds Information

Name of Hunting Ground

[ ]

Number of Hunting Area

- ○ 01

Number of Hunting Ground

[ ]

District

- ○ Placeholder

Municipality

- ○ Placeholder

City/place

[ ]

User of the Hunting Ground (Name of Company)

Address of Hunting Ground

Company ID Number

Responsible person of hunting ground users

Phone number of hunting ground users (Example: XXXXXXXXX)

e-mail address

Hunting ground GPS

Photo of Hunting Ground

1: Hunting Grounds Data

1.1: Are expert hunting management activities carried out by a person licensed to perform these activities in accordance with the Game and Hunting Act (hereinafter: the Law)?

○ Yes

○ No

1.2: Are hunting activities in the hunting area performed by the prescribed number of persons licensed to perform these activities, in accordance with the Law?

○ Yes

○ No

1.2a: If YES, indicate how many persons (if NO, write 0)

1.3: Does the user of the hunting ground manage only one hunting area?

○ Yes

○ No

1.3a: If NO, indicate with which other hunting grounds it manages?

1.3b: If NO, are personnel, vehicles, equipment or facilities shared between the hunting grounds? (If YES, skip question).

○ YES

○ NO

1.4: Does the hunting ground have the hunting base document in accordance with the Law?

○ Yes

○ No

1.5: From what date was the hunting base document brought?

1.5a: Until what date is the hunting base document valid?

1.6: Does the hunting ground have the annual management plan in accordance with the Law?

- Yes
- No

1.7: Does the hunting ground user regularly settle all of his financial obligations?

- Yes
- No

1.8: If the hunting ground user is a hunting association, please indicate the number of members. (Write 0 if none).

1.8a: Indicate the number of members of the association who either own (backyard) or are employed in a pig farm? (Write 0 if none).

1.9: Are the number of members (age, sex, and working-age structure) sufficient for a uniform search of all hunting productive areas for wild boars in the hunting ground, according to the search plan provided by the hunting ground user?

- Yes
- No

1.10: Type of hunting ground

- Mostly Flatland
- Mostly Mountain

1.11: What is the size of the hunting ground (in hectares)?

1.11a: What is the size of the open part of the hunting ground (in hectares)?

1.11b: What is the size of the fenced part of the hunting ground (in hectares)?

1.11c: What percentage of the total area of the hunting ground are forests?

1.11d: What percentage of the total area of the hunting ground are permanent water areas?

1.11e: What percentage of the total area of the hunting ground is agricultural land?

1.12: Does the hunting ground user manage wild boars in the hunting grounds?

- ○ Yes
- ○ No

1.12a: If YES, indicate the number ESTABLISHED by the annual management plan at the beginning of the hunting year. (If NO, write 0).

1.12b: How many are established for the open part? (If none, write 0)

1.12c: How many are established for the fenced part? (If none, write 0)

1.12d: If YES, indicate the number of wild boars PLANNED TO BE HUNTED according to the annual management plan for the current hunting year. (If NO, write 0).

1.12e: How many are planned to be hunted in the open part? (If none, write 0).

1.12f: How many are planned to be hunted in the fenced part? (If none, write 0).

1.12g: If YES, indicate the number of wild boars that have been HUNTED in the hunting ground in the current hunting year. (If NO, write 0).

1.12h: How many have been hunted in the open part? (If none, write 0).

1.12i: How many have been hunted in the fenced part? (If none, write 0).

1.12j: Are wild boars present in the hunting area as transient, occasional or seasonal game during the hunting year?

- ○ Yes
- ○ No

1.13: How large (in hectares) is the Hunting Productive Area (HPA) for wild boar?

1.13a: How large (in hectares) is the HPA for wild boar in the Open Part?

1.13b: How large (in hectares) is the HPA for wild boar in the Fenced Part?

1.14: Indicate other large game managed by the hunting user in accordance with the planning document. (If NO, skip question).

1.15: Were wild boars from other hunting grounds introduced into the hunting grounds within the last 12 months?

- ○ Yes
- ○ No

1.15a: If YES, indicate where the wild boars came from. (If NO, skip question).

1.15b: If YES, were there supporting documents? (If NO, skip question).

- Yes
- No

1.16: Have wild boars been moved out from the hunting ground to other hunting grounds in the last 12 months?

- Yes
- No

1.16a: If YES, indicate where the wild boars were moved to. (If NO, skip question).

1.16b: If YES, were there supporting documents? (If NO, skip question).

- Yes
- No

1.17: Is wild boar hunting practiced by driven hunting?

- Yes
- No

1.18: Is wild boar hunting practiced by individual hunting?

- Yes
- No

1.19: Does the hunting ground have a facility for temporary game meat storage?

- Yes

○ No

1.19a: If YES, indicate the registration number of the facility. (If NO, skip question).

1.19b: If YES, is the facility capable of long term storage of deep-frozen game meat? (If NO, skip question).

○ Yes

○ No

1.19c: If YES, does the facility perform separate storage of game species? (If NO, skip question).

○ Yes

○ No

1.19d: If YES, is the facility located within the hunting grounds? (If NO, skip question).

○ Yes

○ No

1.19e: If YES, what are the geocoordinates of the facility? (If NO, skip question).

1.20: Does the facility temporarily store the game from neighboring hunting grounds?

○ Yes

○ No

1.20a: If YES, indicate from which neighboring hunting grounds. (If NO, skip question).

1.21: Does the hunting ground use a facility from nearby hunting grounds?

- Yes
- No

1.21a: If YES, indicate from which neighboring hunting grounds. (If NO, skip question).

1.22: Does the hunting user own his technically correct vehicles?

- Yes
- No

1.22a: If YES, indicate how many and which type. (If NO, skip question).

2: Status of the Hunting Ground in Respect of ASF Risk Zone

2.1: Depending on the ASF Risk zone, which area is the hunting ground located in?

- ○ Moderate Risk Zone
- ○ High-Risk Zone
- ○ Endangered Area
- ○ Infected Area

2.2: Is the hunting ground established on the border area of the Republic of Serbia or the administrative line?

- ○ Yes
- ○ No

2.2a: If YES, indicate which country or Kosovo*. (If NO, skip the question).

- ○ Bosnia and Herzegovina
- ○ Bulgaria
- ○ Croatia
- ○ Hungary
- ○ Kosovo*
- ○ Montenegro
- ○ North Macedonia
- ○ Romania

2.3: Does the hunting area border the highway/regional road or do the roads pass through the hunting area?

- ○ Yes
- ○ No

2.3a: If YES, does the hunting ground border the highway/regional road or pass through the hunting ground? (If NO, skip the question).

- ○ Hunting ground borders highway/regional road

- ○ Highway/regional road passes through hunting ground

2.4: Is the hunting ground bordered by narrow city are (without suburban settlements) which have the status of an area outside the hunting area, in accordance with the Law?

- ○ Yes
- ○ No

2.5: Is the hunting ground in contact with navigable watercourses?

- ○ Yes
- ○ No

2.6: Is there a commercial catering/tourist facility on the hunting grounds?

- ○ Yes
- ○ No

2.7: Are free-ranging domestic pigs present on the hunting grounds?

- ○ Yes
- ○ No

2.8: Is there a backyard of farm containing pigs within the hunting grounds?

- ○ Yes
- ○ No

3: Conducting Passive and Active Surveillance on the Presence of ASF

3.1: Are wild boars in the hunting ground sampled according to the monitoring plan for Classical and African Swine Fever for the current year?

○ Yes

○ No

3.2: Does the hunting ground have equipment for sampling?

○ Yes

○ No

3:2a: If YES, please specify type and quantity (impermeable bags of 1-3 litres, plastic packaging, vacutainer, knife, portable mobile cooler, ect.). (If NO, skip question).

3.2b: If YES, is the equipment used? (If NO, skip question).

○ Yes

○ No

3.3: Is sampling of hunted wild boars provided in the hunting grounds?

○ Yes

○ No

3.3a: If YES, indicate the number of sampled wild boar during the current hunting year. (If NO, write 0).

3.3b: If YES, indicate the number of sampled wild boar during the past hunting year. (If NO, write 0).

3.4: In accordance with veterinary regulations, have persons engaged in professional hunting activities, or game wardens in the hunting grounds, been trained in the knowledge of game pathology and handling of game and game meat?

- ○ Yes
- ○ No

3.5: Who provides sampling of hunted wild boar in the hunting grounds?

- ○ Trained person from professional or hunting service of hunting ground user, in accordance with veterinary regulations
- ○ Veterinarian-competent veterinary station/clinic
- ○ Veterinary Inspector
- ○ Epizootiologist-competent veterinary institute

3.6: Are wild boars hunted in sanitary hunting in the hunting grounds?

- ○ Yes
- ○ No

3.6a: If YES, indicate the number of wild boar hunted in sanitary hunting during the current hunting year. (If NO, write 0).

[ ]

3.6b: If YES, indicate the number of wild boar hunted in sanitary hunting during the past hunting year. (If NO, write 0).

[ ]

3.6c: If YES, are sanitary hunted wild boars reported to the competent veterinary inspector? (If NO, skip question).

- ○ Yes
- ○ No

3.7: Is the hunting grounds being searched for wild boar carcasses? (If NO, skip question).

- ○ Yes
- ○ No

3.7a: If YES, indicate the number of wild boar carcasses found during the current hunting year. (If NO, write 0).

3.7b: If YES, indicate the number of wild boar carcasses found during the past hunting year. (If NO, write 0).

3.8: Are wild boar carcasses reported to the competent veterinary service/inspection?

- ○ Yes
- ○ No

3.8a: If YES, indicate the number of wild boar carcasses reported during the current hunting year. (If NO, write 0).

3.8b: If YES, indicate the number of wild boar carcasses reported during the past hunting year. (If NO, write 0).

3.9: Are found wild boar carcasses sampled?

- ○ Yes
- ○ No

3.9a: If YES, indicate the number of wild boar carcasses sampled during the current hunting year. (If NO, write 0).

3.9b: If YES, indicate the number of wild boar carcasses sampled during the past hunting year. (If NO, write 0).

3.10: Are found wild boar carcasses sampled on the spot?

- Yes
- No

3.11: Has a hunting grounds map been created for carcass searching?

- Yes
- No

3.12: Are written records about searching kept?

- Yes
- No

3.13: Is a written form for the sample completed during each sampling

- Yes
- No

3.14: For hunting grounds located in the high risk area (the Republic of Romania or Bulgaria), has the hunting ground user conducted during the 2018/19 hunting season the reduction of the wild boar population in accordance with the issued instructions?

- Yes
- No

4: Control of the Wild Boar Hunting Procedure

4.1: Does the hunting ground user report the next hunt to the

veterinary inspection or local authority veterinary station at least 48 hours before hunting?

- ○ Yes
- ○ No

4.2: Does the hunting ground user obtain a statement for all persons involved in hunting about previous hunting activities within the past 30 days?

- ○ Yes
- ○ No

4.3: Does the hunting ground user prohibit access to hunting grounds for persons who have visited other hunting grounds or pig farms within the past 72 hours?

- ○ Yes
- ○ No

4.4: Does the hunting ground user prohibit the bringing in of meat products without a declaration into the hunting grounds?

- ○ Yes
- ○ No

4.5: Is the removal of internal organs of hunted wild boar carried out on the spot?

- ○ Yes
- ○ No

4.5a: If YES, are the collected intestines transported to the temporary game meat storage facility? (If NO, skip question).

- ○ Yes
- ○ No

4.5b: If YES, are the collected intestines packaged and transported in a sealed package without leaks and drops? (If NO, skip question).

- Yes
- No

4.5c: If YES, are the collected intestines packaged separately from each individual? (If NO, skip question).

- Yes
- No

4.6: Is the evisceration of hunted wild boar carried out at a collection point within the hunting ground?

- Yes
- No

4.6a: If YES, are the collected intestines transported to the temporary game meat storage facility? (If NO, skip question).

- Yes
- No

4.6b: If YES, are the collected intestines packaged and transported in a sealed package without leaks and drops? (If NO, skip question).

- Yes
- No

4.6c: If YES, are the collected intestines packaged separately from each individual? (If NO, skip question).

- Yes
- No

4.6d: If YES, how many collection points are there within the hunting grounds? (If, NO, write 0).

4.6e: If YES, indicate geographic coordinates of ALL collection points. (If NO, skip question).

4.7: Is the evisceration of hunted wild boar carried out within the temporary game meat storage facility?

- Yes
- No

4.7a: If YES, are the collected intestines packaged in a sealed package without leaks and drops? (If NO, skip question).

- Yes
- No

4.7b: If YES, are the collected intestines packaged separately from each individual? (If NO, skip question).

- Yes
- No

4.8: Does the hunting ground user use official marks with numbers for marking the big game?

- Yes
- No

4.8a: If YES, are marks used for marking each hunted wild boar? (If NO, skip question).

- Yes
- No

4.8b: If YES, are marks used for marking an individual packing with intestines? (If NO, skip question).

- Yes

- ○ No

4.9: Does the veterinary inspector or local authority veterinarian perform official control of big game?

- ○ Yes
- ○ No

4.10: Before being placed on the market, is every hunted wild boar carcass accompanied by a certificate of safety of meat and organs of game for human consumption?

- ○ Yes
- ○ No

4.11: Is wild boar meat from regular hunting placed on the market before receiving negative laboratory results of diagnostic testing for an infectious disease, like ASF?

- ○ Yes
- ○ No

4.12: Is wild boar meat from sanitary hunting placed on the market before receiving negative laboratory results of diagnostic testing for an infectious disease, like ASF?

- ○ Yes
- ○ No

4.13: Are intestines kept until negative laboratory results of a diagnostic test for an infectious disease, like ASF, is received?

- ○ Yes
- ○ No

4.14: Are wild boar trophies placed on the market before receiving negative laboratory results of diagnostic testing for an infectious disease, like ASF?

- ○ Yes
- ○ No

4.15: Are there records of sending materials for laboratory testing?

- Yes
- No

4.16: Are there records of laboratory test results?

- Yes
- No

4.17: Are there records of game meat buyers?

- Yes
- No

4.18: Are hunting dogs allowed in hunting?

- Yes
- No

4.19: Are dogs without the owner, or without the owner's control, removed from the hunting ground?

- Yes
- No

5: Disinfection Measures

5.1: Are appropriate disinfectants used with a good term of validity?

- Yes
- No

5.1a: If YES, which current stock is available? (If NO, skip question).

5.2: Is disinfectant used according to the manufacturer instructions?

- ○ Yes
- ○ No

5.2a: If YES, are there using records? (If NO, skip question).

- ○ Yes
- ○ No

5.3: Are there records of the conducted cleaning and disinfection measures?

- ○ Yes
- ○ No

5.4: Are the outside evisceration sites disinfected after the evisceration has been carried out?

- ○ Yes
- ○ No

5.5: Within the hunting ground, are own official vehicles the only vehicles used?

- ○ Yes
- ○ No

5.6: Are vehicles cleaned and disinfected after each transportation?

- ○ Yes
- ○ No

5.7: Are temporary storage equipment and facilities cleaned and disinfected after each use?

- ○ Yes
- ○ No

5.8: Are disinfectant barriers present on the hunting grounds?

- ○ Yes
- ○ No

5.8a: If YES, indicate how many? (If NO, write 0).

5.8b: If YES, indicate where they are? (If NO, skip question).

5.8c: If YES, are they used regularly? (If NO, skip question).

- ○ Yes
- ○ No

5.9: Is there equipment for field disinfection in the hunting ground?

- ○ Yes
- ○ No

5.9a: If YES, specify type and quantity of equipment (hand sprayers, machine sprayers, washing brushes, water hoses, hand bins, disinfectant preparation drums, vehicle and pedestrian sponges, nylon..) (If NO, skip question).

5.9b: If YES, are they used regularly? (If NO, skip question).

- ○ Yes
- ○ No

5.10: Is there biosafety protective equipment available?

- ○ Yes
- ○ No

5.10a: If YES, specify type and quantity of equipment (disposable suits, protective reusable gloves, disposable gloves, disposable shoe covers, safety goggles..) (If NO, skip question).

5.10b: If YES, are they used regularly? (If NO, skip question).

- ○ Yes
- ○ No

5.11: Do licensed employees have protective equipment (professional hunters)?

- ○ Yes
- ○ No

5.11a: If YES, do they have: (If NO, skip question).

- ☐ A pair of work suits

- [ ] Two pairs of work shoes
- [ ] Two pairs of work boots

5.11b: If YES, are they used regularly? (If NO, skip question).

- ( ) Yes
- ( ) No

6: Control of the Procedure for the Safe Removal of Offals or Dead Animals

6.1: Which of the following methods are used to dispose offal (from hunted animals)?

- [ ] Leaving on the field
- [ ] Burial on the spot
- [ ] Burial in a pit
- [ ] Burning
- [ ] Rending

6.2: Which of the following methods are used to dispose found dead wild boar carcasses?

- [ ] Leaving on the field
- [ ] Burial on the spot
- [ ] Burial in a pit
- [ ] Burning
- [ ] Rending

6.3: Does the hunting ground have a container for the offals and dead animals?

- ( ) Yes
- ( ) No

6.3a: If YES, indicate how many (If NO, write 0).

6.3b: If YES, is it regularly used? (If NO, skip question)

- Yes
- No

6.3c: If YES, does the hunting ground have a contract with the authorized company for removing offals or dead animals? (If NO, skip question)

- Yes
- No

6.3d: If YES, are there records? (If NO, skip question)

- Yes
- No

6.3e: How often does the User of the hunting ground use an authorized company to dispose found dead wild boar?

- Always
- Often
- Rarely
- Never

6.4: Is there a pit for dead wild boar inside the hunting ground?

- Yes
- No

6.4a: If YES, is it regularly used? (If NO, skip question)

- Yes
- No

6.4b: If YES, is it registered and approved from authorities? (If NO, skip question)

- Yes
- No

6.4c: If YES, are there records of use? (If NO, skip question)

- Yes
- No

6.4d: If YES, indicate the coordinates for all. (If NO, skip question).

6.4e: How often does the User of the hunting ground use pits (located on the hunting ground) to dispose found dead wild boar?

- Always
- Often
- Rarely
- Never

6.5: Does the practice of burning dead wild boars occur?

- Yes
- No

6.5a: If YES, does burning occur at the pit site? (If NO, skip question).

- Yes
- No

6.5b: If YES, indicate the coordinates for all. (If NO, skip question).

6.5c: How often does the User of the hunting ground use burning to dispose found dead wild boar?

- ○ Always
- ○ Often
- ○ Rarely
- ○ Never

6.6: Do persons disinfect their hands, clothing, footwear, and equipment after manipulation or handling of offals or dead animals?

- ○ Yes
- ○ No

6.7: Are there designated sites for the new pits in the case of the appearance of an infectious disease, like ASF?

- ○ Yes
- ○ No

6.7a: If YES, indicate how many (If NO, write 0).

6.7b: If YES, indicate the coordinates for all. (If NO, skip question).

6.8: Is there a working cargo transport machine within the hunting ground?

- Yes
- No

6.8a: If YES, specify type and quantity (tractor, trailer, tractor basket,...) (If NO, skip question).

6.8b: If YES, are they regularly used? (If NO, skip question).

- Yes
- No

6.9: Is there an own working machine for land digging within the hunting ground?

- Yes
- No

6.9a: If YES, specify type and quantity (excavator, bucket tractor,...) (If NO, skip question).

6.9b: If YES, are they regularly used? (If NO, skip question).

- Yes

○ No

6.10: Are there hand tools for land digging within the hunting ground?

○ Yes

○ No

6.10a: If YES, specify type and quantity (spade, shovel, pick, wheelbarrow,...) (If NO, skip question).

6.10b: If YES, are they regularly used? (If NO, skip question).

○ Yes

○ No

6.11: Within the hunting ground, are there leak-proof packaging for packing and transporting carcasses without leaks and drips?

○ Yes

○ No

6.11a: If YES, specify type and quantity (waterproof bags, plastic baths/container for transport, rope for binding,...) (If NO, skip question).

6.11b: If YES, are they regularly used? (If NO, skip question).

- ○ Yes
- ○ No

7: Feed Ban Control

7.1: Does the hunting ground user practice wild boar supplementary feeding?

- ○ Yes
- ○ No

7.2: Is supplementary feeding carried out with animal food with controlled origin?

- ○ Yes
- ○ No

7.3: Does the hunting ground user use swill in wild boar feeding?

- ○ Yes
- ○ No

7.4: Do wild boars have access to landfills?

- ○ Yes
- ○ No

7.5: Do wild boars have access to scavenging places?

- ○ Yes
- ○ No

7.6: Is there a record of wild boar supplementary feeding?

- ○ Yes
- ○ No

8. Awareness raising

8.1: Did the hunting ground user put out posters to raise awareness among visitors to the hunting ground, to report dead wild boar carcasses?

- ○ Yes
- ○ No

8.1a: IF YES, did visitors to the hunting ground report found dead wild boar carcasses in the past 12 months?

- ○ Yes
- ○ No

8.2: Did the hunting ground user put out any other posters to raise awareness among visitors to the hunting ground, on how they can prevent introduction or spread of ASF?

- ○ Yes
- ○ No

8.2a: IF YES, what was the aim of the poster(s)?

- ○ Prevent introduction of ASF into the hunting ground
- ○ Prevent spread of ASF from the (infected) hunting ground
- ○ Both
- ○ Neither

8.3: Did the hunting ground user inform its member on how to prevent the introduction and spread of ASF?

- ○ Yes
- ○ No

9: Previous Controls

9.1: Has the veterinary or forest-hunting inspectors ordered any measures in the past 2 years?

- Yes
- No

9.1a: If YES, have they been implemented?

- Yes
- No

9.1b: If YES, was the documentation kept? (If NO, skip question).

- Yes
- No

10: Feasibility study

When answering feasibility questions, take into account your current human and financial resources, equipment and facilities available, costs of implementation, current practices and habits.

10.1: How feasible is it that Hunting ground management does NOT use kitchen/restaurant waste or other sources of animal protein (swill) to feed wild boar?

- Impossible
- Very difficult
- Difficult
- Possible with some effort
- Possible with little effort

Possible with no effort

10.2: How feasible is it to create a plan on how and where to dispose of dead wild boar in case of an ASF outbreak?

- Impossible
- Very difficult
- Difficult
- Possible with some effort
- Possible with little effort
- Possible with no effort

10.3: How feasible is it to NOT to transport live wild boars into or outside of your hunting ground?

- Impossible
- Very difficult
- Difficult
- Possible with some effort
- Possible with little effort
- Possible with no effort

10.4: How feasible is it to decrease the density of wild boar by increasing hunting, eliminating supplementary feeding or other means?

- Impossible
- Very difficult
- Difficult
- Possible with some effort
- Possible with little effort
- Possible with no effort

10.5: How feasible is it to prevent wild boar accessing landfills or scavenging places?

- Impossible
- Very difficult
- Difficult
- Possible with some effort
- Possible with little effort
- Possible with no effort

10.6: How feasible is it to have leak-proof bags or tanks available on stock for transport dead wild boar carcasses out from the hunting ground?

- Impossible
- Very difficult
- Difficult
- Possible with some effort
- Possible with little effort
- Possible with no effort

10.7: How feasible is it to dispose of all or most found dead wild boar?

- Impossible
- Very difficult
- Difficult
- Possible with some effort
- Possible with little effort
- Possible with no effort

10.8: How feasible is it to inform the public and hunters (through posters, flyers or face-to-face briefings) about the

importance to report dead wild boar and about the main ASF prevention measures?

- Impossible
- Very difficult
- Difficult
- Possible with some effort
- Possible with little effort
- Possible with no effort

10.9: How feasible is it to forbid visitors and hunters from bringing meat products into the HG's HPA?

- Impossible
- Very difficult
- Difficult
- Possible with some effort
- Possible with little effort
- Possible with no effort

10.10: How feasible is it to destroy all offal from hunted animals?

- Impossible
- Very difficult
- Difficult
- Possible with some effort
- Possible with little effort
- Possible with no effort

10.11: How feasible is it NOT to share any personnel, vehicles, equipment or facilities (like dressing or storage area) with other hunting grounds?

- Impossible
- Very difficult
- Difficult
- Possible with some effort
- Possible with little effort
- Possible with no effort

10.12: How feasible is it to always clean and disinfect the hands, footwear, clothing and equipment after dressing and/or handling dead animals?

- Impossible
- Very difficult
- Difficult
- Possible with some effort
- Possible with little effort
- Possible with no effort

10.13: How feasible is it to always clean and disinfect vehicles before they leave the hunting ground?

- Impossible
- Very difficult
- Difficult
- Possible with some effort
- Possible with little effort
- Possible with no effort

10.14: How feasible is it to forbid from hunting those hunters who own pigs or work on pig farms?

- Impossible
- Very difficult

- Difficult
- Possible with some effort
- Possible with little effort
- Possible with no effort

10.15: How feasible is it to forbid driven hunts?

- Impossible
- Very difficult
- Difficult
- Possible with some effort
- Possible with little effort
- Possible with no effort

10.16: How feasible is it to forbid hunters who have visited pig farms in the last 72 hours from hunting?

- Impossible
- Very difficult
- Difficult
- Possible with some effort
- Possible with little effort
- Possible with no effort

10.17: How feasible is it to always clean and disinfect hunting equipment, facilities, meat storage or dressing areas after they are used?

- Impossible
- Very difficult
- Difficult
- Possible with some effort

- ○ Possible with little effort
- ○ Possible with no effort

10.18: How feasible is it to establish fixed areas to dress carcasses?

- ○ Impossible
- ○ Very difficult
- ○ Difficult
- ○ Possible with some effort
- ○ Possible with little effort
- ○ Possible with no effort

Place of filling in form

Date of Survey

Responsible Person

