# Supplementary material for: A Novel Tool to Assess the Risk for African Swine Fever in Hunting Environments: The Balkan Experience
Source: Pathogens. 2022 Dec 3;11(12):1466. doi: 10.3390/pathogens11121466 (PMC9787848; doi:10.3390/pathogens11121466)
Supplement: Supplementary file 1 [file pathogens-11-01466-s001.zip › Supplementary File S3.pdf]

Hunting ground activities and biosecurity measures for African swine fever control in Serbia.

Orrico M, Beltran-Alcrudo D, Hovari M

Given the importance of wild boar in the spread of African swine fever (ASF), the assessment of features, practices and existing biosecurity in hunting grounds is essential to understand (and eventually reduce) the risk of disease incursions and further spread and to improve early detection, i.e. the finding of ASF-positive wild boar soon after the virus is introduced.

With this questionnaire, we would like your help to quantify the importance of the major risk factors related to ASF in hunting grounds. These risk factors will help us to evaluate the likelihood of three outcomes: 1) the risk of ASF introduction into a hunting ground; 2) the risk of ASF spread within the hunting ground or from one hunting ground into another; and 3) the capacity to detect ASF in a hunting ground.

Instruction for scoring system.

0 *=not relevant;* ***1*** *= negligible;* ***2*** *= low;* ***3*** *= medium;* ***4*** *= high;* ***5*** *= critical*

Do not score shaded cells.

If you do not feel comfortable with an answer, please use the cell "comments"

When assessing the risk of spread, the model only considers spread from hunting grounds into other hunting grounds, but not into domestic pig holdings.

This model is only fully applicable to hunting grounds that are already affected or at a medium or high risk of ASF introduction, i.e. within already infected countries or close (or linked) to infected countries. So when filling in the tables, please answer keeping only such hunting grounds in mind.

Acronyms and definitions.

FENCED AREA : Area of land fenced with natural or artificial obstacles to prevent or reduce the movement of game.

HG : Hunting ground. Defined as a natural area in which there are conditions for permanent protection, management, hunting, use, and improvement of game populations.

HPA: Hunting productive area. Defined as part of the hunting ground where the hunting species of the game has favorable conditions for life, reproduction, proper development, and constant survival. (When we refer to HPA in this questionnaire it is always intended as HPA for wild boar.)

CULLING: Shooting of wild boar (sick, injured or for disease management) with the intention of collecting and destroying the whole carcass (no dressing takes place).

USER: The entity that uses the hunting ground. Could be a hunting association, public enterprise, national park, etc.

WB: Wild boar

General information

Please provide the following information before completing the questionnaire. Your answers will remain anonymous.

*Surname:
*First name:
*Department, Institution, Country:
*Position held:
*Field of expertise (veterinarian,
*Telephone number:
*E-mail address:
*Other information:

SECTION 1: Hunting Ground Characteristics

Hunting grounds (HG) characteristics play a fundamental role in the epidemiology of ASF. In this section, we refer to the characteristics of the hunting ground

Using a scale from 0 to 5, please indicate the importance of each factor to increase each of the risks .

Risk Factors	Risk of ASF introduction into hunting ground	Risk of ASF spread to other hunting grounds	Probability of ASF detection in hunting ground	Comments
High WB density in the HPA				
Large size of the HPA				
Low proportion of fenced area within the HPA				
WB being transient (occasional or seasonal game)				
HPA is in contact (i.e. crossed or bordered) with roads and/or navigable watercourses.				
HPA is in contact with cities and settlements				

SECTION 2: Management Plan

Having a management plan is a mandatory legal requirement and its content can affect the epidemiology of ASF on the HG.

Using a scale from 0 to 5, please indicate the importance of each factor to increase each of the risks .

Risk Factors	Risk of ASF introduction into hunting ground	Risk of ASF spread to other hunting grounds	Probability of ASF detection in hunting ground	Comments
HG not collecting information on the last hunting activity of hunters				
Movements of live WB between HGs. (This refers only to WB moved/transported by humans)				
Shared personnel vehicles, equipment and facilities (e.g.: dressing and storage areas) with other hunting ground/s				
Absence of transport and/or storage space used only for WB meat in order to avoid cross contamination of meat from other species.				
Driven hunt is practiced				
High number of visitors into the HG				
Absence of fixed (designated) dressing areas				

SECTION 3: Domestic pig - wild boar interface

The interaction between domestic and wild boar populations is considered a route of transmission for ASF. Direct contact between the populations, environmental contamination or ingestion of contaminated material (e.g. food waste, feed, garbage or pig carcasses) is relevant for ASF epidemiology.

Using a scale from 0 to 5, please indicate the importance of each factor to increase each of the risks .

Risk Factors	Risk of ASF introduction into hunting ground	Risk of ASF spread to other hunting grounds	Probability of ASF detection in hunting ground	Comments
High number of pigs in low biosecurity farms (e.g.: free range or backyard) present in HG				
Hunters who own pigs or work on pigs farms.				
People who visited other				

hunting grounds or pig farms in the last 72 hours are allowed to hunt.				
Bringing meat products into the HPA is allowed				

SECTION 4: Awareness

Knowledge of ASF, how to recognize it, and the basic measures to prevent its transmission is important to detect, prevent and control the disease. This is important not only for the hunters, but also for other leisure activities conducted in wild boar habitats

Using a scale from 0 to 5, please indicate the importance of each factor to increase each of the risks .

Risk Factors	Risk of ASF introduction into hunting ground	Risk of ASF spread to other hunting grounds	Probability of ASF detection in hunting ground	Comments
Absence of posters/ flyers/briefings to inform the public and hunters upon entering HPA about the importance of ASF and measures to prevent it				
Absence of posters/ flyers/briefings to inform the public and hunters about reporting dead WB in the HPA				

SECTION 5 : Passive and Active surveillance performed

Carcass reporting and testing of dead/shot animals is critical for early detection and represents one of the most important activities where hunters are directly involved.

Using a scale from 0 to 5, please indicate the importance of each factor to increase each of the risks .

Risk Factors	Risk of ASF introduction into hunting ground	Risk of ASF spread to other hunting grounds	Probability of ASF detection in hunting ground	Comments
Sampling of hunted WB for ASF testing				
Active search for dead WB carcasses				
Low Effort/incentive in finding dead WB carcasses in the HG				
Training of the HG manager in game pathology and handling of game and game meat				
Absence of culling (as per definition written in the introduction)				
Meat or trophies leaving the hunting ground without an ASF PCR negative test				

SECTION 6: Cleaning and Disinfection Procedures

Cleaning and disinfection procedures adopted during hunting, the transport of game and the disposal of offal or dead animals are essential to reduce environmental contamination with ASF virus. Not following disinfection procedures increases the risk of introduction into the hunting ground but also the risk of further spread because boots, clothes, rifles, vehicles, etc. can become contaminated and spread the virus to other hunting grounds.

Using a scale from 0 to 5, please indicate the importance of each factor to increase each of the risks .

Risk Factors	Risk of ASF introduction into hunting ground	Risk of ASF spread to other hunting grounds	Probability of ASF detection in hunting ground	Comments
Absence of disinfectants and cleaning and disinfection procedures for equipment				

procedures for equipment, facilities, meat storage, evisceration sites.				
Absence of disinfection point for every visitor				
Absence of disinfection barriers				
Absence of cleaning and disinfection of hands, footwear, clothing and personal equipment after offal and manipulation of dead animals				
Vehicles used for hunting that leave the HG without being cleaned and disinfected first				

SECTION 7: Management aspects for disposal of offal and found dead wild boar

The proper disposal of offal and found dead wild boar is important to reduce the spread of ASF because it will decrease the virus load in the environment. Not following disposal procedures is increasing the risk of further spread because the higher the virus load the higher the probability of spreading the disease either through natural movement of infected wild boar or through contaminated boots, clothes, rifles, vehicles, etc.

Using a scale from 0 to 5, please indicate the importance of each factor to increase each of the risks .

Risk Factors	Risk of ASF introduction into hunting ground	Risk of ASF spread to other hunting grounds	Probability of ASF detection in hunting ground	Comments
No systematic destruction of offal (from hunted animals)				
Limited disposal of found dead WB				
Absence of a disposal plan in case of an ASF outbreak				
Absence of cargo transport or leak-proof bags/tanks to move dead WB				

SECTION 8: Feeding and implementation of measures

Supplementary feeding influences the reproduction rate, density, the behaviour and movement patterns of wild boar and consequently the risk of ASF introduction and spread. It may also bring wild boar together to the feeding sites and attract wild boar from other areas. Furthermore, feed used poses a risk if it is infected with ASF virus. The implementation of measures instructed by the veterinary authorities in the past is an indicator of the stakeholder's involvement and reliability.

Using a scale from 0 to 5, please indicate the importance of each factor to increase each of the risks .

Risk Factors	Risk of ASF introduction into hunting ground	Risk of ASF spread to other hunting grounds	Probability of ASF detection in hunting ground	Comments
Supplementary feeding of WB (Note: consider only impact on behaviour)				
Supplementary feeding of WB with products of animal origin from uncontrolled sources (swill or kitchen waste)				
Access to landfills or scavenging places for WB				
Lack of compliance by the HG user i.e. the user has not applied measures ordered by the authorities in the past 2 years				
