# Supplementary material for: A Novel Tool to Assess the Risk for African Swine Fever in Hunting Environments: The Balkan Experience
Source: Pathogens. 2022 Dec 3;11(12):1466. doi: 10.3390/pathogens11121466 (PMC9787848; doi:10.3390/pathogens11121466)
Supplement: Supplementary file 1 [file pathogens-11-01466-s001.zip › Supplementary file S4.pdf]

Supplementary Material File S4

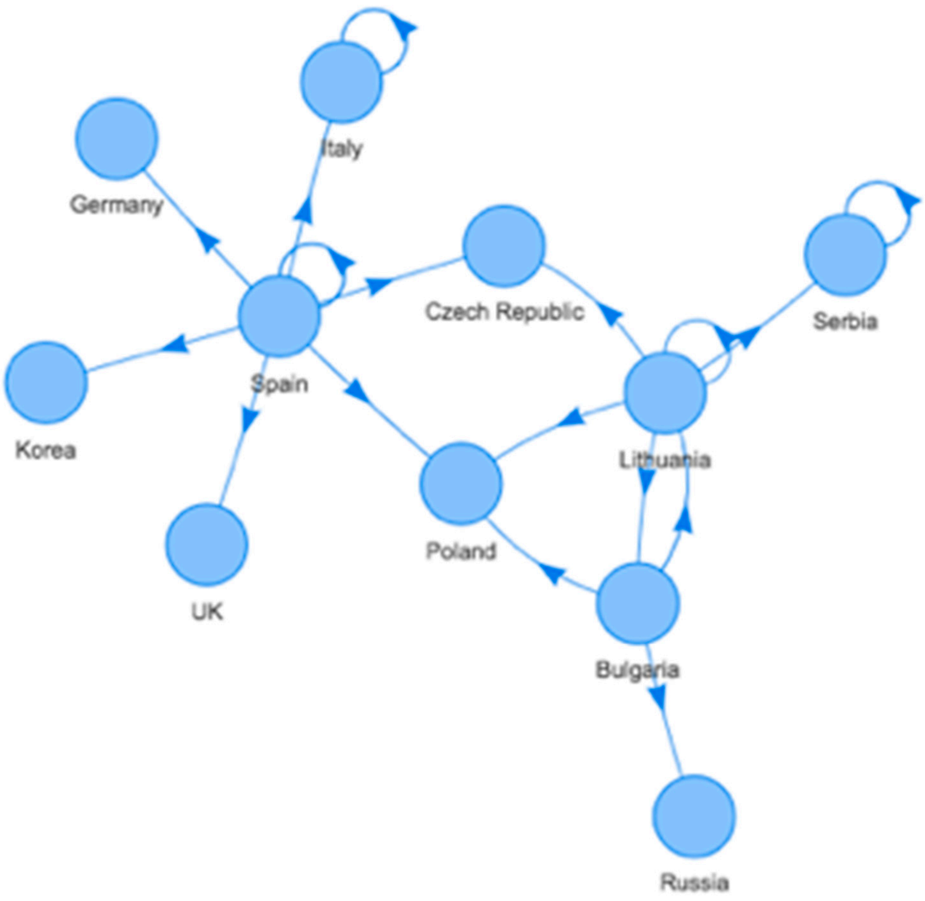

Figure S1 – Network countries analysis of the experts in the first wave

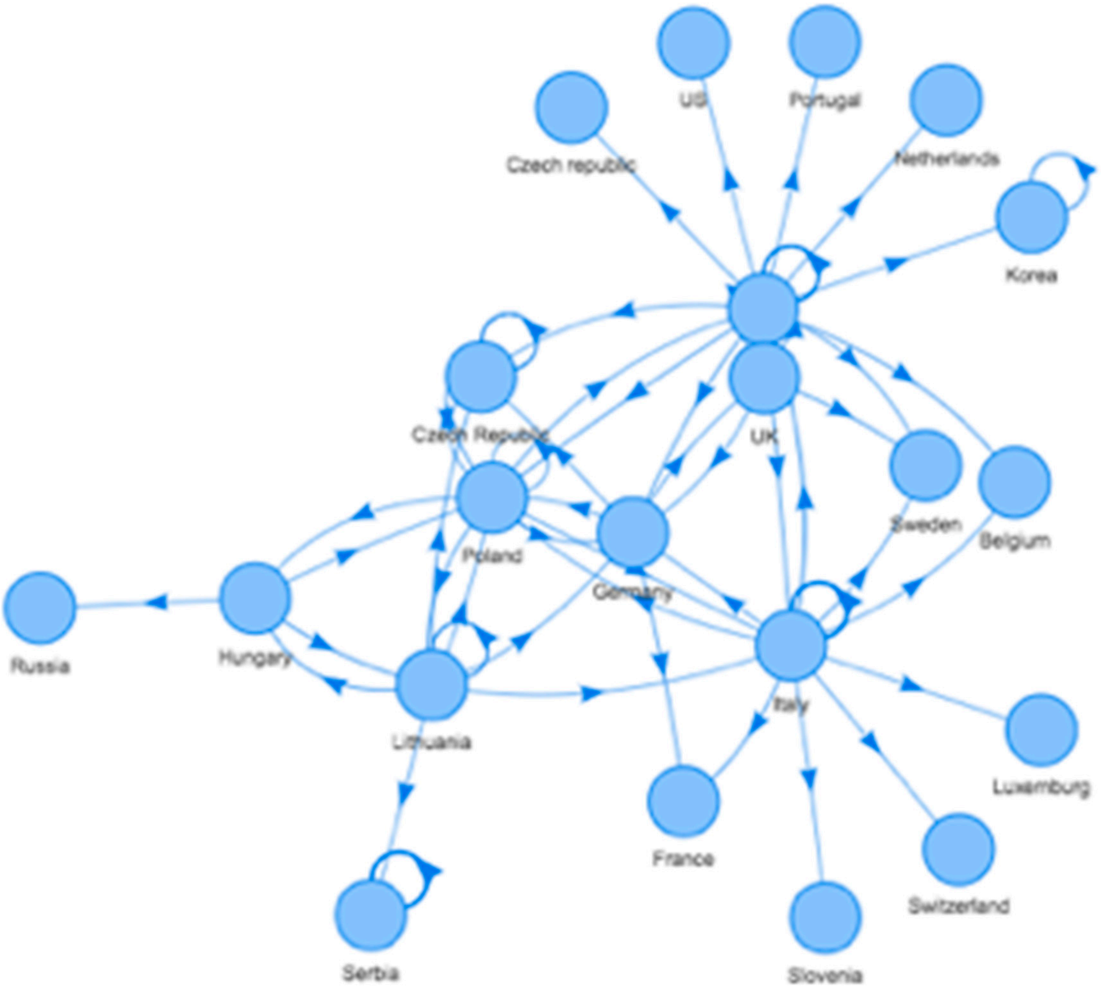

Figure S2 – Network countries analysis of the experts in the second wave

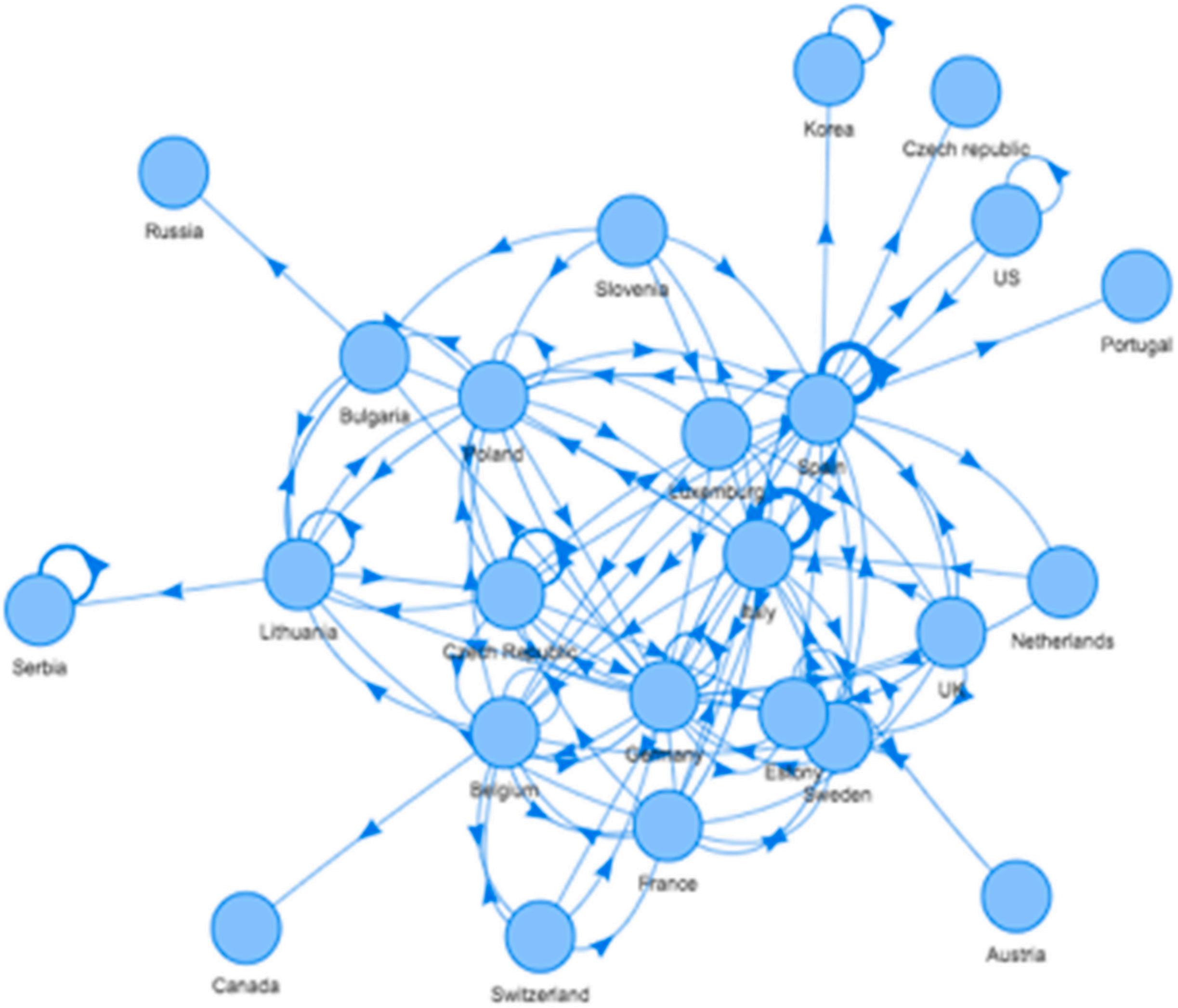

Figure S3 – Network countries analysis of the experts in the third wave.
