# Supplementary material for: A Novel Tool to Assess the Risk for African Swine Fever in Hunting Environments: The Balkan Experience
Source: Pathogens. 2022 Dec 3;11(12):1466. doi: 10.3390/pathogens11121466 (PMC9787848; doi:10.3390/pathogens11121466)
Supplement: Supplementary file 1 [file pathogens-11-01466-s001.zip › Supplementary File S5.pdf]

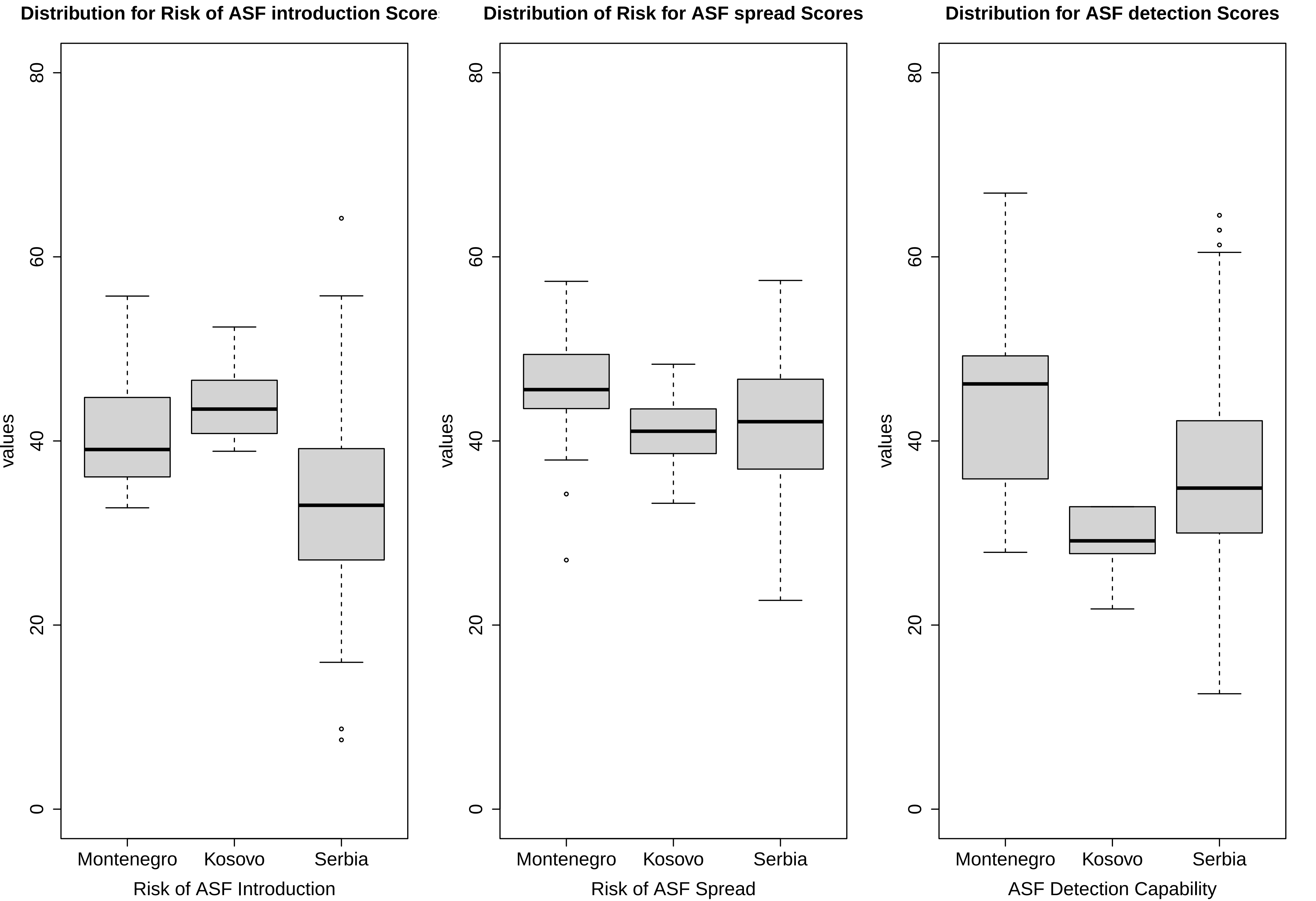

Distribution for Risk of ASF introduction Scores
Distribution of Risk for ASF spread Scores
Distribution for ASF detection Scores
values
0
20
40
60
80
Montenegro
Kosovo
Serbia
Risk of ASF Introduction
Risk of ASF Spread
ASF Detection Capability
