# Supplementary material for: A Novel Tool to Assess the Risk for African Swine Fever in Hunting Environments: The Balkan Experience
Source: Pathogens. 2022 Dec 3;11(12):1466. doi: 10.3390/pathogens11121466 (PMC9787848; doi:10.3390/pathogens11121466)
Supplement: Supplementary file 1 [file pathogens-11-01466-s001.zip › Supplementary file S6.pdf]

Answers of feasibility questions by hunting ground managers

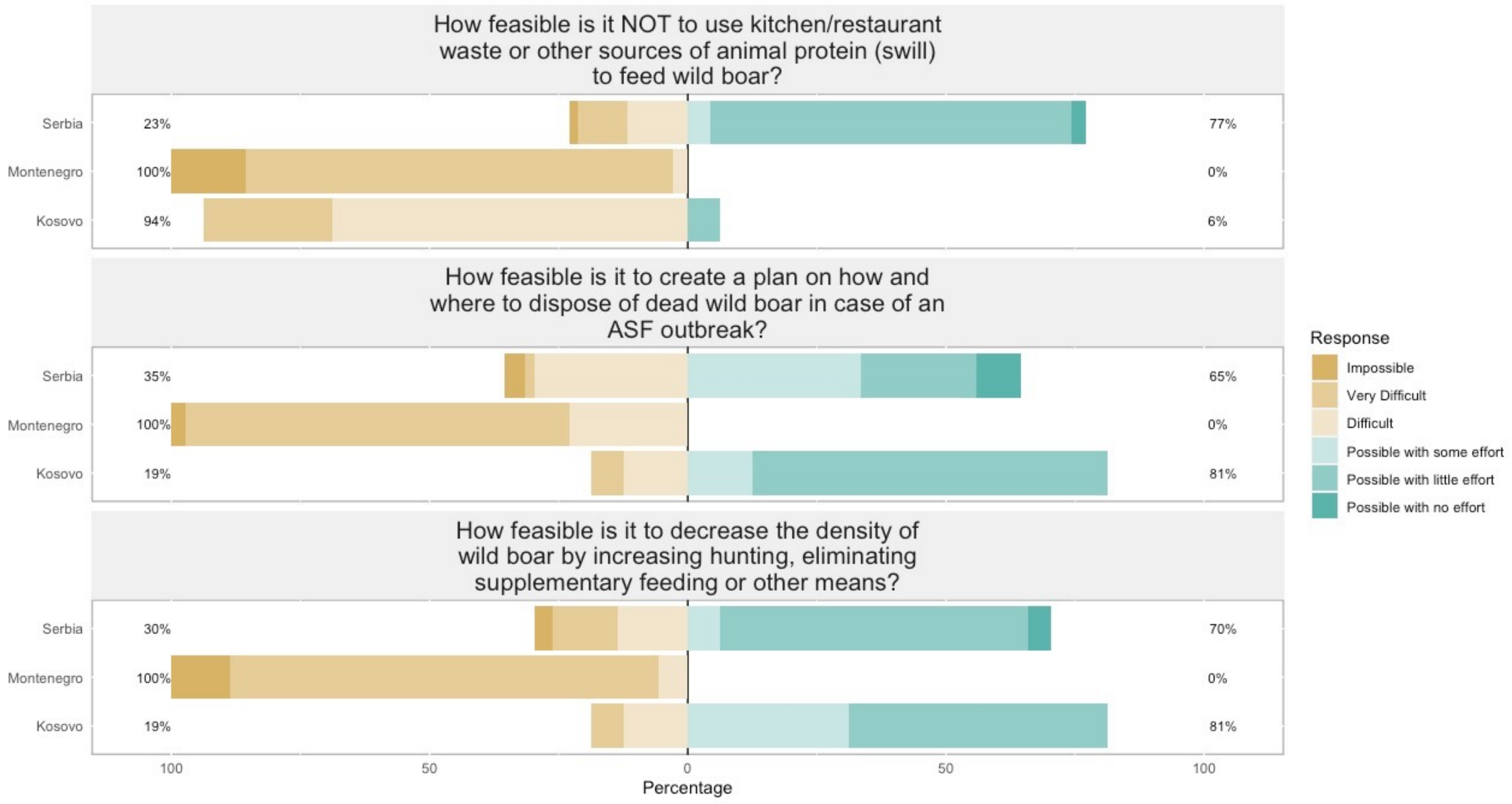

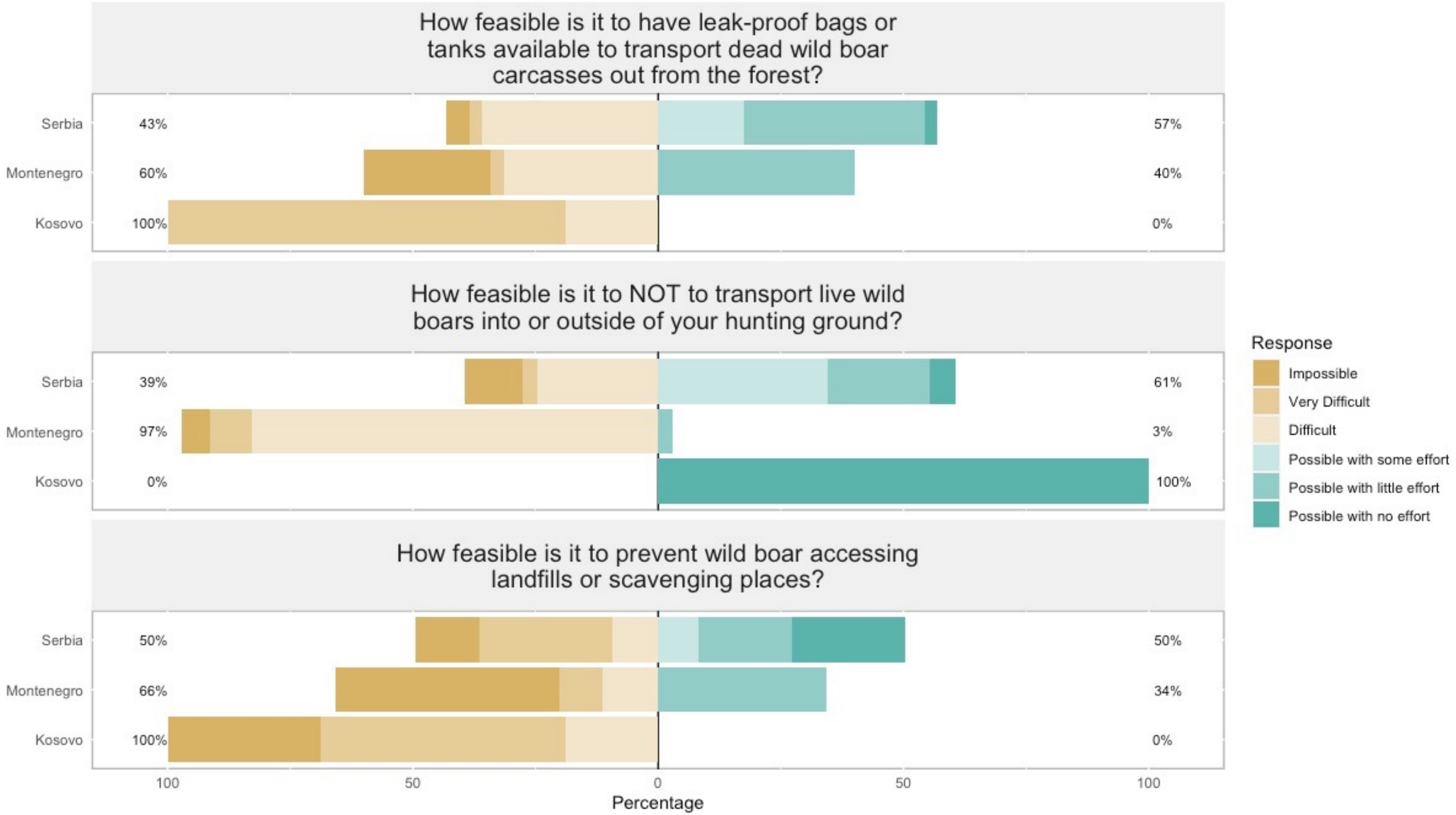
How feasible is it to have leak-proof bags or tanks available to transport dead wild boar carcasses out from the forest?
Serbia
43%
57%
Montenegro
60%
40%
Kosovo
100%
0%
How feasible is it to NOT to transport live wild boars into or outside of your hunting ground?
Serbia
39%
61%
Montenegro
97%
3%
Kosovo
0%
100%
How feasible is it to prevent wild boar accessing landfills or scavenging places?
Serbia
50%
50%
Montenegro
66%
34%
Kosovo
100%
0%
100
50
0
50
100
Percentage
Response
Impossible
Very Difficult
Difficult
Possible with some effort
Possible with little effort
Possible with no effort

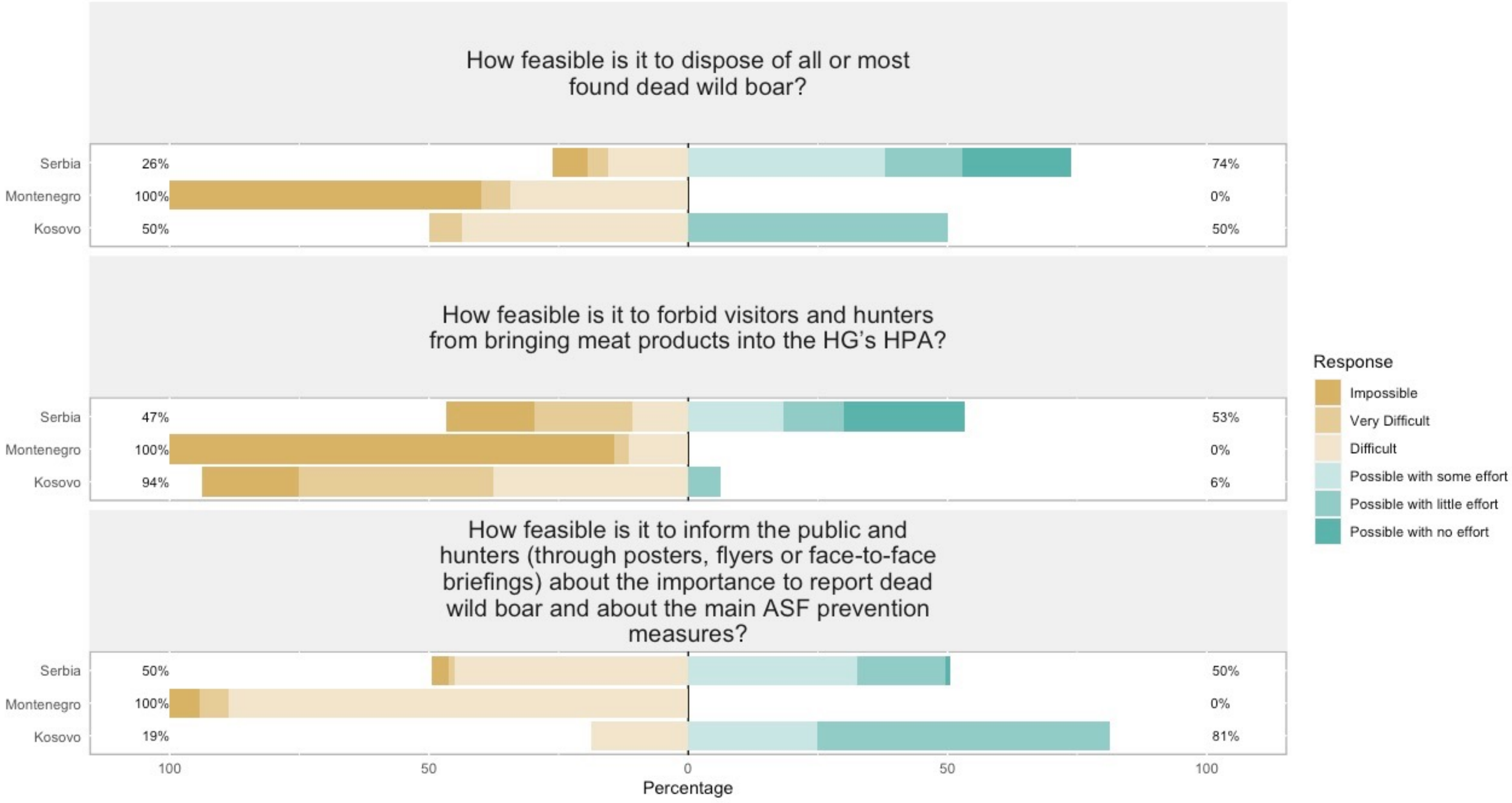
How feasible is it to dispose of all or most found dead wild boar?
Serbia 26% 74%
Montenegro 100% 0%
Kosovo 50% 50%
How feasible is it to forbid visitors and hunters from bringing meat products into the HG's HPA?
Serbia 47% 53%
Montenegro 100% 0%
Kosovo 94% 6%
How feasible is it to inform the public and hunters (through posters, flyers or face-to-face briefings) about the importance to report dead wild boar and about the main ASF prevention measures?
Serbia 50% 50%
Montenegro 100% 0%
Kosovo 19% 81%
100
50
0
50
100
Percentage
Response
Impossible
Very Difficult
Difficult
Possible with some effort
Possible with little effort
Possible with no effort

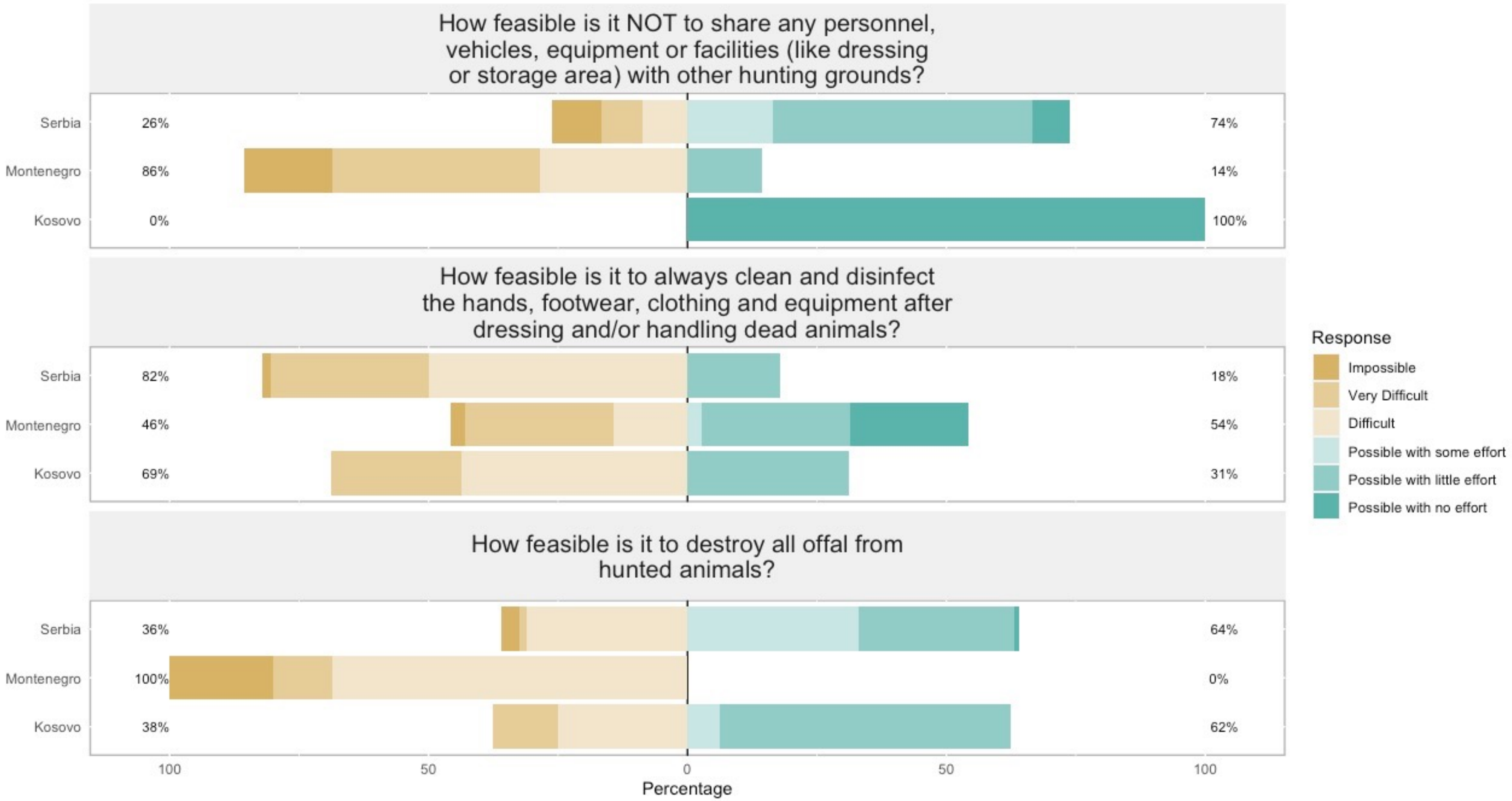
How feasible is it NOT to share any personnel, vehicles, equipment or facilities (like dressing or storage area) with other hunting grounds?
Serbia
26%
74%
Montenegro
86%
14%
Kosovo
0%
100%
How feasible is it to always clean and disinfect the hands, footwear, clothing and equipment after dressing and/or handling dead animals?
Serbia
82%
18%
Montenegro
46%
54%
Kosovo
69%
31%
How feasible is it to destroy all offal from hunted animals?
Serbia
36%
64%
Montenegro
100%
0%
Kosovo
38%
62%
100
50
0
50
100
Percentage
Response
Impossible
Very Difficult
Difficult
Possible with some effort
Possible with little effort
Possible with no effort

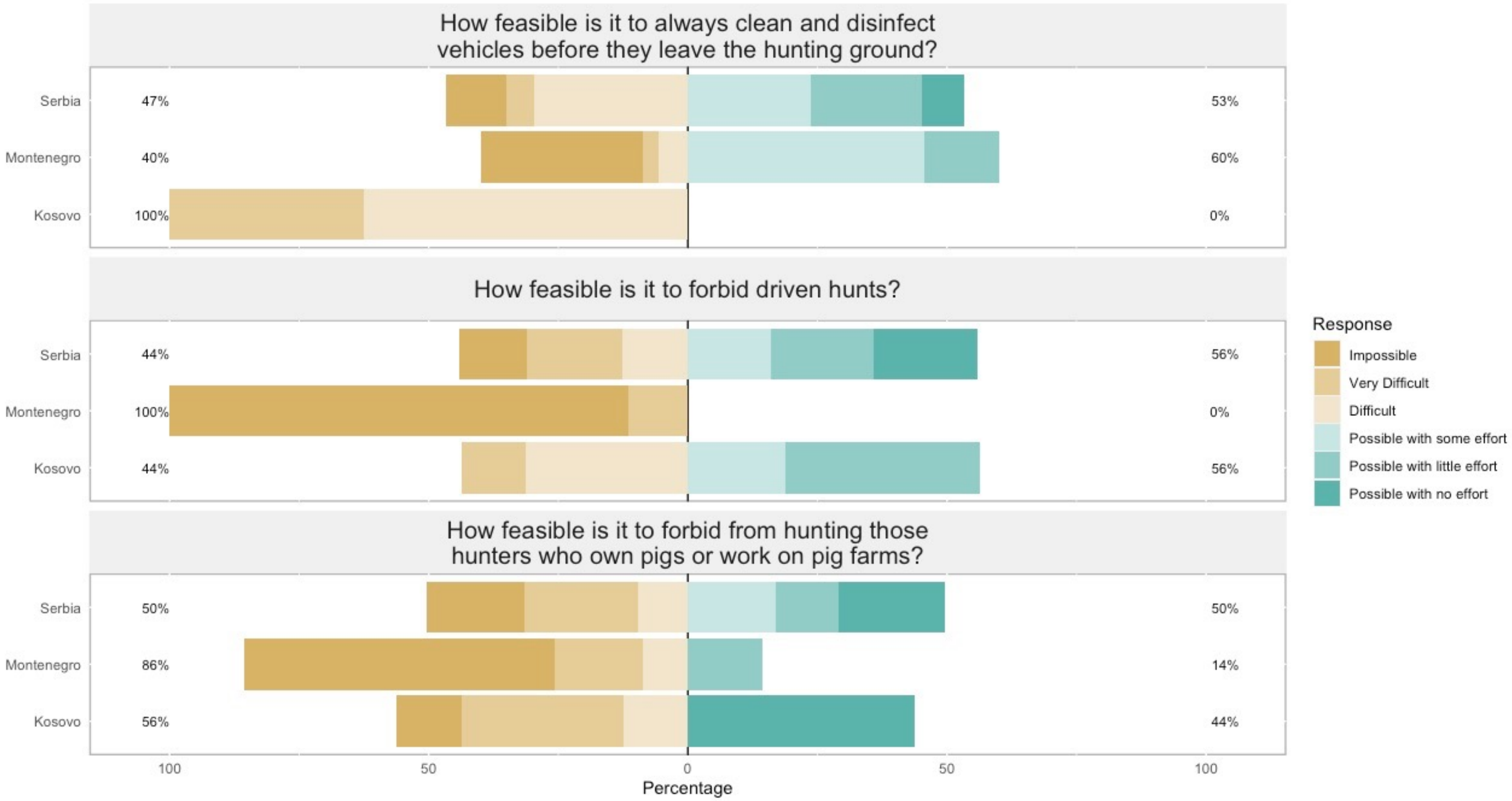
How feasible is it to always clean and disinfect vehicles before they leave the hunting ground?
Serbia
47%
53%
Montenegro
40%
60%
Kosovo
100%
0%
How feasible is it to forbid driven hunts?
Serbia
44%
56%
Montenegro
100%
0%
Kosovo
44%
56%
How feasible is it to forbid from hunting those hunters who own pigs or work on pig farms?
Serbia
50%
50%
Montenegro
86%
14%
Kosovo
56%
44%
100
50
0
50
100
Percentage
Response
Impossible
Very Difficult
Difficult
Possible with some effort
Possible with little effort
Possible with no effort

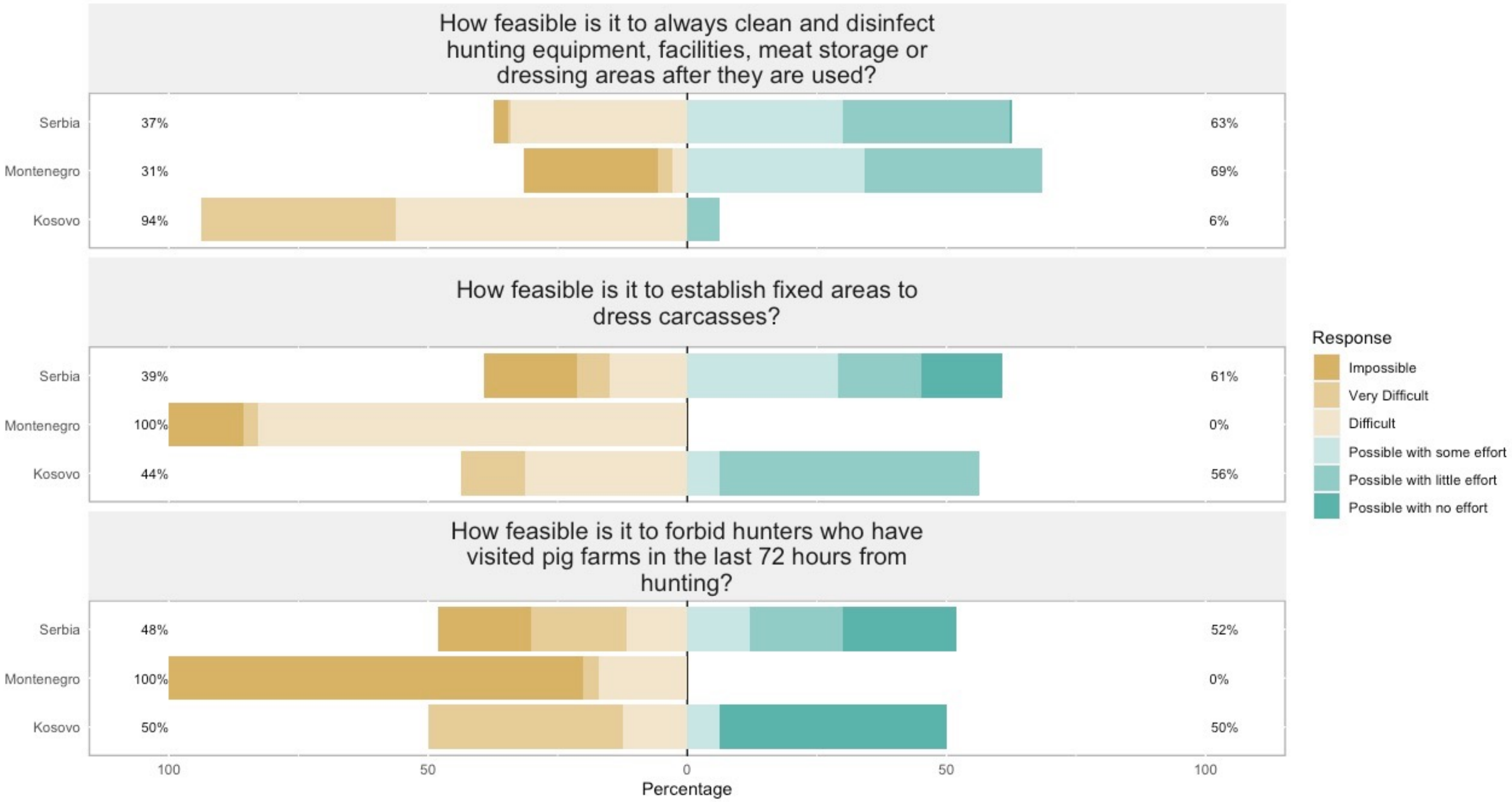
How feasible is it to always clean and disinfect hunting equipment, facilities, meat storage or dressing areas after they are used?
Serbia 37% 63%
Montenegro 31% 69%
Kosovo 94% 6%
How feasible is it to establish fixed areas to dress carcasses?
Serbia 39% 61%
Montenegro 100% 0%
Kosovo 44% 56%
How feasible is it to forbid hunters who have visited pig farms in the last 72 hours from hunting?
Serbia 48% 52%
Montenegro 100% 0%
Kosovo 50% 50%
100
50
0
50
100
Percentage
Response
Impossible
Very Difficult
Difficult
Possible with some effort
Possible with little effort
Possible with no effort
