# Supplementary material for: A Novel Tool to Assess the Risk for African Swine Fever in Hunting Environments: The Balkan Experience
Source: Pathogens. 2022 Dec 3;11(12):1466. doi: 10.3390/pathogens11121466 (PMC9787848; doi:10.3390/pathogens11121466)

Supplementary File S7 - Maps showing the resulting risk following the introduction of mitigation measures of low, medium and high effort

Low effort intervention | Medium effort intervention | High effort intervention

Rows: Risk of ASF Introduction; Risk of ASF Spread; Overall ASF Risk

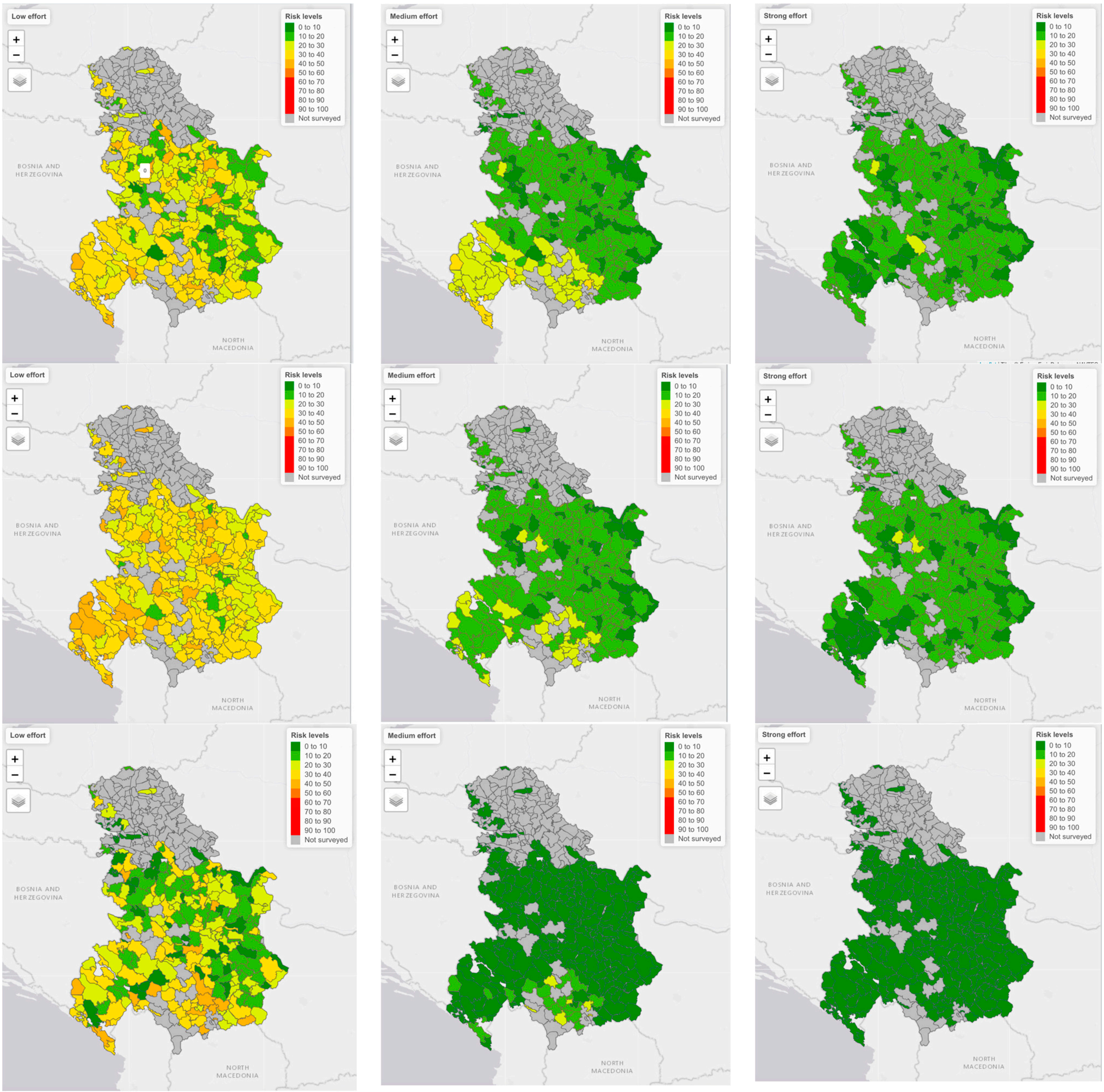

Supplement: Supplementary file 1 [file pathogens-11-01466-s001.zip › Supplementary file S7.pdf]
